# Standards - the common element in providing the safety, quality and performance of the medical practice

**Published:** 2009

**Authors:** Mihai Greabu

**Affiliations:** * Department of Biochemistry, Faculty of Dental Medicine „Carol Davila” University of Medicine and Pharmacy, Bucharest

**Keywords:** standards, medicine, dentistry, health

## Abstract

Knowing and applying standards is an opportunity of the years 2007-2008 in any kind of field where a successful activity is intended and this assures a certain way towards competence and quality.

The most recent German studies highlighted, to the surprise of the specialists, that standardization holds the second place, after the material means, in the row of the elements considered to be decisive for the success of a business. The existence of standards and the concern for their implementation in the activity provides a high technical and quality level of the products services offered to the clients and the increase in the level of competence of the personnel, who will be able to cope with all the challenges. This need comes from the process of Romania’s accession to the European Union.

There are a lot of reasons why standards represent a fundamental part of our daily life. Practically, we are surrounded by standards. Everything is „working” well and it is efficient if the standards used as a base for manufacturing „things” have been correctly developed and applied.

Standards open communication channels and commercial channels, promote the understanding of technical products, the compatibility of products and services, facilitate mass production and, most importantly, they are the necessary base for the achievement of the objectives in the fields of health and safety and a better quality of life.

The transition towards the global market needs an instrument for the removal of the barriers to the application of the latest discoveries in the field of medical instruments, materials and manual labor.

Each medical device, equipment and material used in the Dental and General Medicine is standardized, in fact that leads to their better knowing and provides controllable treatment for manual labor with predictable and repeatable results.

This presentation intends to make a survey of some general aspects on standardization as well as a review of the standards in the medical area.

Standards belong to the quotidian existence because they can be found in almost everything around us. There are multiple reasons for standards to represent a fundamental part of our daily life. Practically, we are surrounded by standards. All of these „works” would have been efficient if the standards used have been applied correctly.

The beginning of standardization was marked two centuries BC when the first Chinese Emperor, Qin Shi Huangdi, introduced unique technical standards in the following domains: the Wheel, the base in transportation, carriages, the width of town gates, and the roads construction, measurements units for lengths and weight, pipes for water, weapons and armors.

The ISO/CEI 2 Guide: 1996 defines the standard as being a document, established by consensus and approved by recognized body, which assures, for common and repeated use, rules, directives or characteristics for activities or their results, aiming to obtain the optimum level of order in a certain context.

A standard is a document that establishes a common language and prescriptions promoting the exchange of goods between a buyer and a seller and protects the general prosperity. Standardization represents a common solution to solve a problem.

## International, regional and national Standardization

Standards are developed at international, regional and national level and the coordination of the activity at these 3 levels is assured by common structures and cooperation agreements.

International Standardization is represented by ISO (International Standardization Organization), IEC (International Electrotechnical Commission), and ITU (International Telecommunications Union).

Regional Standardization includes CEN, The European Committee for Standardization, CENELEC, and The European Committee for Electrotechnical Standardization, ETSI, and The European Institute for Standardization in Telecommunications, America, COPANT, Pan American Standardization Commission and MERCOSUR, Common Market of the South.

Concerning national standardization, every country has its own national body for standardization. The national bodies can be members of regional or international bodies.

At a national level, the standardization activity is developed in technical committees for standardization that can benefit from assistance given by the experts’ group. The committees are set up by experts representing industry, research institutes, universities, public authorities, consumers or other profile bodies.

The activity is led by technical committees, where secretariats are under responsibility of national bodies and all national members have the right to be represented in International or regional committees for each domain separately.

## Standardization and World Trade Organization (WTO)

The latest negotiations of GATT (The General Agreement over Prices, Taxes and Commerce), the Uruguay Round, took place as a result of the WTO setting up (January 1st, 1995). The agreement related to Technical Barriers to Trade (WTO/TBT), is one of these 29 individual judicial texts of WTO agreement, making it mandatory for the members to ensure the fact the technical rules, the standards and procedures of conformity assessment do not create futile obstacles in the way of commerce. Annex 3 of the TBT Agreement is the Code of Good Practice for Preparing, Adopting and Application of Standards. By accepting the TBT Agreement, the WTO members agreed to assure the fact that the governments and the standardization bodies of their countries, accept and respect this Code of Good Practice and accept to take reasonable measures that the local governments, the NGO’s (Non-Governmental Organizations), and the regional standardization bodies do the same thing.

The TBT Agreement recognizes the important contribution of international standards and the system for the assessment and endorsement which can result in an increase in efficiency and can facilitate international commerce.

Standards:

- open communication and commercial channels;

- promote the understanding of technical products;

- assure the compatibility of products and services;

- facilitate the mass production;

­- develop the necessary base in order to attain the objectives of health, security and a better quality of life.

Standardization is recognized today as the essential discipline for all the economical factors; we mostly make efforts to know their motivation and implication and a cooperating modality for the safety, quality and performance of the medical practice. In the year 2000, a study of DIN (Deutsches Institut für Normung) evidenced that the second factor which contributes to the economical increase, after capital, is represented by standards.

***The influencing factors*** are:

1. Economical Integration of Europe

The rapid steps to European economical integration and the decision of the CE Commission to give to standards a decisive relation with the free circulation of goods and services within the Union, led to the key role as a normative instrument. The increasing competition and specialization which are determined by standards will lead to a major development of the exchange in the Single Market. These exchanges would have to conform to certain rules. The Commission limited the role by stating the objectives – essential needs – leaving the economical factor, involved in standards developing, the possibilities to specify the modalities and the means of reaching the objectives.

2. Quality

It came out in the 50’s and gained an increased importance and it is evidently, more and more, a driving factor of competition. If today it is easy to compare prices, it is much difficult to compare the quality of levels. The existence of a reference quality system, unanimously recognized, represents a precious clarification of an instrument. This is exactly the standards’ role.

3. The technical and technological evolution

Conformity assessment against standards is more important for quality assurance. 

Certification is the procedure by which a third party gives a written assurance that a product, process or service is in conformity with requirements. (Definition: Guide 2 ISO/CEI: 1996). It is different from other systems due to the process of establishing the conformity, like the manufacturers declarations of conformity, testing laboratories, analytical reports or of inspection bodies, based on the results of tests inspections and audits and will give the client confidence in the systematic intervention of a third competent party.

How are the info instruments? Due to the fact that an increased number of standards catalogues are structured in conformity with ICS (International Classification of Standards), the users of standards benefit from a common access key to the world standards. The users get a larger image over their own domain of interest due to a multiple classification of standards and are better guided during the study. International standards classification includes 40 domains, divided into 390 groups, which are divided in 895 subgroups.

Using the European and international standards adopted by Romania is one of the most important conditions for Romania’s European Integration. Standardization and European Integration come out as the application of the principle of mutual recognition. The voluntary use of standards introduces those that do it, in the big family of those using the standards, offering a consumption market and a life of products up to the limit of adopted standards system. The application of a national standards system opens only for the national market, the European Standards system opens the European market and an international standards system opens the international market. The globalization concept is based on voluntary application of standards, of technical regulations and of procedures for the evaluation of the identical or compatible factor aiming to conformity.

The voluntary character of standards application concept was determined by necessity of avoiding the technical barriers to trade, keeping a reasonable rhythm among the technical progress and the introduction of the results in national and international standards.

The New Approach defines the essential requirements through the European Directives standing for the very general concepts which could be practically applied by respecting one or several standards. Presumptions of conformity of a product with the essential requirements in the Directives constitute one of the reasons which it is necessary to use harmonized standards for.

What do Harmonized European Standards represent? They are standards by developed European Standardization bodies and published in the Official Journal of the European Community, conferring the presumption of conformity with the essential requirements of a certain European Directive.

***CEN/TC 251*** working on the prevention or elimination of differences with technical content, from the standards which have the same scope, especially, those differences which can constitute barriers to free circulation.

***ISO/TC 215*** signed a cooperation agreement with those in CEN/TC 251, for the harmonization of standards in the medical domain.

International standards are the global market instrument. The transition to a global market needs an instrument for the removal of technical barriers from the way of the application of the newest discoveries in the medical instrumentation, materials and the works field. ISO and CEI support the principles „A standard, a test, a conformity assessment procedure” or „Once tested, accepted everywhere”.

The conformity with the harmonized standards is the simplest way to abide by the law and technical regulations. 

The product conformity assessment consists in measures taken by the producers’ client, regulatory authorities and independent, third party, to evaluate the conformity related to a reference document and depend on the existence of a standard or another reference document to compare the assessed product.

The benefits of using national standards adopting European and International standards are:

- clients’ increased confidence in products and services manufactured and sold in conformity with European and International standards;

- increased competition of products and services; 

- avoiding the multiple verifications regarding conformity assessment in the regulated areas;

- promoting the Romanian products on the Single European market.

On the other hand, Romania, as a CEN/CENELEC and ETSI member, through its national body for standardization – The Romanian Standards Association - ASRO, had to adopt European Standards as national standards. A European Standard can be made available to the Romanian users only by national adoption. Only one official version of a standard is allowed to exist in the same language and translation of European or International standards, representing an exclusive right of ASRO. 

CEN/CENELEC Guide Part 2 presents common rules for standards adoption. An EN will be adopted as national standards, by translation or by identically publishing the text in one of the official languages – English, French or German. Another rule is to avoid any standard national conflict and to solve any technical conflict. An EN will be adopted by identically taking the text and presenting the standard without application restriction.

The CEN/CENELEC type of Publications are:

EN European Standard

Adoption under the same format. Existent national standards in conflict with EN must be withdrawn.

HD Harmonized Document

The subject must be adopted and existent national standards in conflict with EN must be withdrawn.

ENV European Pre standard 

Adoption under the same format. ENV does not need the withdrawal of the conflicting national standards.

CR CEN/CENELEC Report

The type of publication used for info documents which has no statute of being a standard.

CWA Seminar on CEN Agreement

EUROPEAN LEGISLATION AND EUROPEAN STANDARDS

During the adoption process of internal legislative market which regulates the free market of goods, the European legislator developed the essential requirements for products related to:

- Security

- Health

- Environment

These are included in the Directives which harmonized the technical regulations. These essential requirements defined by the European legislator are then detailed in the European Harmonized Standards developed by the CEN, CENELEC and ETSI, the European Standardization bodies.

The harmonization of the national regulations with the Community ones is materialized by:

- the correspondence between the European and Romanian Directive document;

- European Directives are adopted on national level by Government Decision

- the European Harmonized Standards with those being nominated by Order of Ministers; 

- national specialized authorities;

- Romanian technical specialized committees;

NEW APPROACH:

- Represents new strategy and techniques

- Includes the *Directives* which establish the essential requirements related to security, health and environment

- Those essential requirements are detailed in the Harmonized European Standards, which offers the presumption of conformity with the Directives

New Approach and Global Approach

In accordance with the New Approach in regulating the products and the Global Approach in evaluating the assessment, the intervention is limited to what is essential and gives a large freedom in choosing the way of accomplishing the public obligations. The correlation between the technical committees for Romanian, European and international Standardization with responsibilities of adopting harmonized standards referring to in vitro medical diagnostic equipment is the following:

The domain of medical instrument is regulated by 3 European Directives: 

- 93/42/CE – Medical Equipment.

- 90/385/CEE – Medical Equipment on active implants.

- 98/79/CE – Medical equipment for *in vitro* diagnoses.

There is a list of Harmonized European Standards in the European Directive 93/42/CEE published in Official Journal of the European Union. There are 260 European Standards included in this list, which were adopted as Romanian Standards. 

The correlation between the technical committees of Romanian Standardization and the European and international ones:

- ***CT 99*** „Biological evaluation and medical equipment including human and/or animals’ cells”, corresponding to CEN/TC 194, 206, 258 and 316

- ***CT 350*** „Quality Management related to medical equipment”, corresponding to CEN/CLC/WG QS, CEN/SS S99 and ISO/TC 210

- ***CT 369*** „Technical helping means nonactive surgical implants”, corresponding to CEN/TC 215, 239, 285, 293 and ISO/TC 121, 150, 168, 170 and 173

- ***CT 370*** „Medical reactive equipment, in vitro diagnostic terminology and symbols”, corresponding to CEN/TC 102, 140, 204, 205, 257, 347 and ISO/TC 76, 84, 157, 198 and 212.

TELEHEALTH - ISO/TC 215-assures the extension of health protection service in the far-way located communities and the integration in a medical system.

SMART CARD - ISO 21549 + EUROCARDS of European Commission.

Due to standardization, a medical doctor from any country will get access to the medical data of a patient. Austria and Germany already introduced the Health Card. It is necessary to solve certain ethical and technical aspects related to the assurance of security and confidentiality of information while referring to the state of a patient.

TECHNICAL COMMITTEES - DENTISTRY

The correspondence between New Directives approaches and Romanian normative documents:

- CT 349 – Dentistry– mirror technical committee CEN/TC 55.

- CT 191 – Equipments, apparatus of technical – medical products – mirror Romanian technical committee for ISO TC 106 – Products and equipment for dentistry.

There are more than 150 standards in the domain of Dental Medicine. Standards represent great benefits for medicine due to many reasons:

- maintaining the state of health, is an important political objective; in all countries the political decisions have a strong impact;

- increase in the exchange of information between the educational medical field and that of research → new solutions and → the orientation in learning to new requirements in the medical domain;

- modern medicine is the beneficiary of a much larger horizon and much more possibilities.

The standardization of the laboratory tests (ISO/TC 212+ CEN/TC 140/WG 4) determine:

- increase the competence and quality of medical laboratories;

- establish a correct and real equilibrium between theory and practice;

- improve the accuracy of quantitative determination methods;

- make it possible for a patient to obtain the same results in any laboratory in the world.

Even so, they bring the smile on our face; the dental standards are a serious matter!

Standards play a vital role in improving and assuring the quality, efficiency and security of materials of the dental work. A simple visit to the dental doctor (dentist) involves more than a dozen of standards beginning with the dental chair, illuminated by a dental light and going on with how to clean, shape and seal the root canal. There are more than 20 ISO dental standards involving definitions, codes and designations, materials and equipment. An ISO designed system shows the location of each tooth in a dental chart and shaping the crown requires a variety of instruments conforming to standards. Moreover, an extensive coding system is used to classify rotary instruments with limits for bore sizes and dimensions for discs, materials, etc. The biocompatibility and corrosion of these materials are evaluated according to standards. Developing dental standards generate an informational flux: companies, dental medics, educational system, and governmental institutions. 

***ISO/TC106***, developing standards coordinating dental practice, has 7 subcommittees (Filling and restorative materials, Prosthodontic materials, Terminology, Dental instruments, Dental equipment, Oral care products and Dental implants) and over 40 working groups. The following are evaluated according to standards: equipment, biocompatibility of dental materials, metals corrosion, renewing of tissues and alveolar bone, the substitutes of alveolar bone in implants, tooth paste, whitening products and others solutions of oral hygiene

***ISO/TC156*** was developed to increase the quality of dental work. It is an important aspect that ~90% of world’s population suffers from a dental affection and a big part uses solutions for oral hygiene. Dental market is distinguished as an extremely dynamic and competitive market. In the developed countries the report dentist: patients is =1:2000...3000, in the developing countries - the report dentist: patients is =1>1000000, and in Romania= 1:7000.

ISO cooperation with EU assures the improvement of the quality, security and services performance and it is illustrated by the fact that 123 ISO standards were accepted and adopted as EN ISO standards by CEN/TC in August 2006. Modern dentistry has a greater scope and more possibilities than in the past. The future is represented by the areas such as tissue engineering and bone growth.

Dental standards and the environment - ISO 11143 - evidenced that it was absolutely necessary to develop a standard amalgam because the pollution with mercury (Hg) from dental offices is estimated to 0,04%-0,2% from the total pollution with Hg in the world.

## Conclusions

* The standardization’s advantages are: improving the conformity of products, processes and services with (precise) destination, the uniform application of used medical procedures, the facilitation of technological co-operation.

* Every equipment, device and material used in medical practice is standardized, determining their knowledge and assuring treatment procedures which can be controllable and with predictable and repeatable results.

* Preoccupation for knowledge and abiding by the regulations, existing in each state or private institution, faculty or professional association, should be done in the same manner thus assuring a high level of standardization applied at European, International or national base level.
